# Paying health workers for performance in Battagram district, Pakistan

**DOI:** 10.1186/1478-4491-9-23

**Published:** 2011-10-07

**Authors:** Sophie Witter, Tehzeeb Zulfiqur, Sarah Javeed, Amanullah Khan, Abdul Bari

**Affiliations:** 1 Oxford Policy Management, Oxford, UK; 2 Queen Margaret University, Edinburgh, UK; 3 Islamabad Office, Oxford Policy Management, Pakistan; 4 Oxford Policy Management, Pakistan; 5 Health & Nutrition, Save the Children US, Pakistan; 6 Save the Children US, Pakistan

## Abstract

**Background:**

There is a growing interest in using pay-for-performance mechanisms in low and middle-income countries in order to improve the performance of health care providers. However, at present there is a dearth of independent evaluations of such approaches which can guide understanding of their potential and risks in differing contexts. This article presents the results of an evaluation of a project managed by an international non-governmental organisation in one district of Pakistan. It aims to contribute to learning about the design and implementation of pay-for-performance systems and their impact on health worker motivation.

**Methods:**

Quantitative analysis was conducted of health management information system (HMIS) data, financial records, and project documents covering the period 2007-2010. Key informant interviews were carried out with stakeholders at all levels. At facility level, in-depth interviews were held, as were focus group discussions with staff and community members.

**Results:**

The wider project in Battagram had contributed to rebuilding district health services at a cost of less than US$4.5 per capita and achieved growth in outputs. Staff, managers and clients were appreciative of the gains in availability and quality of services. However, the role that the performance-based incentive (PBI) component played was less clear--PBI formed a relatively small component of pay, and did not increase in line with outputs. There was little evidence from interviews and data that the conditional element of the PBIs influenced behaviour. They were appreciated as a top-up to pay, but remained low in relative terms, and only slightly and indirectly related to individual performance. Moreover, they were implemented independently of the wider health system and presented a clear challenge for longer term integration and sustainability.

**Conclusions:**

Challenges for performance-based pay approaches include the balance of rewarding individual versus team efforts; reflecting process and outcome indicators; judging the right level of incentives; allowing for very different starting points and situations; designing a system which is simple enough for participants to comprehend; and the tension between independent monitoring and integration in a national system. Further documentation of process and cost-effectiveness, and careful examination of the wider impacts of paying for performance, are still needed.

## Background

Improving the performance of health care delivery systems is an important objective, both in high-income settings but even more critically in low- and middle-income settings, where resources for health are much more constrained.

Pay-for-performance is currently receiving increased attention as a strategy for improving the performance of healthcare providers, organisations and governments. It is also promoted as an important tool for achieving the health Millennium Development Goals, and for improving the effectiveness of development aid. However, there is currently a lack of rigorous evidence on the effectiveness of these strategies in improving health care and health, particularly in lower income countries [Witter et al, Paying providers for performance in health care in low and middle income countries: a systematic review, submitted to Cochrane Collaboration, 2011; [1,2]].

Pay-for-performance refers to the transfer of money or material goods conditional on taking a measurable action or achieving a predetermined performance target [3]. While paying for performance is relatively a simple concept, it includes a wide range of interventions that vary with respect to the level at which the incentives are targeted (recipients of healthcare, individual providers of healthcare, health care facilities, private sector organizations, public sector organizations and national or sub-national levels). The types of outputs or outcomes targeted can also vary widely, as can the type of accompanying measures (such as investments in training, equipment and overall resources).

In OECD countries, paying for performance is generally described as a tool for improving quality [4]. In low and middle income countries, however, it generally has wider objectives [Witter et al, Paying providers for performance in health care in low and middle income countries: a systematic review, submitted to Cochrane Collaboration, 2011], including:

• to increase the allocation efficiency of health services (by encouraging the provision of high priority and cost effective services)

• to increase the technical efficiency (by making better use of existing resources such as health staff)

• to improve equity of outcomes (for example, by encouraging expansion of services to hard-to-reach groups)

Independent evaluations of pay-for-performance schemes--their design, implementation and cost-effectiveness--are important to inform the policy debate about the different modalities of paying for performance and their likely contribution in different contexts. They also contribute to the wider discussion of the relative role of financial and non-financial incentives in motivating health worker [6,7].

This article aims to contribute to published experiences of paying providers for performance in low-income settings, based on an independent review of a district-based pay-for-performance health project in Pakistan.

### The project

Save the Children US (SC US) started working in Battagram district, North-West Frontier Province, Pakistan, after the earthquake of 8 October 2005. Battagram has a total land area of 1301 square kilometres. The estimated population of Battagram in 2004-2005 was 361 000, with 277 inhabitants per square kilometre. In April 2008, following the initial emergency and relief phase, SC US entered a public-private partnership to revitalise primary health care in the district through reconstruction, equipment, provision of supplies, management support and training.

The project was funded by the World Bank and Japan International Cooperation Agency with an overall budget of just under $3 million. It was planned for a period of two years, ending in June 2010.

The district health system in Pakistan is composed of two tiers of public healthcare facilities. The primary health care services are provided at dispensaries, basic health units (BHUs) and rural health centres (RHCs). Secondary care--including first and second referral facilities providing acute, ambulatory and inpatient care--are provided through Tehsil and district headquarter hospitals (DHQs). An important feature of the project was that the provincial government agreed to transfer the district health budget to the Save the Children account. Save the Children was authorized to organize and manage the healthcare services (including human resource management, and maintenance of health facilities); procure and supply medicines; implement the health management information system; and monitor and supervise the health system in Battagram.

As part of project implementation the district was divided into four 'hubs', centred around the rural health centres. The hub centres acted as referral facilities for the attached basic health centres, civil dispensaries, maternal and child health centres and tuberculosis control centres located in their catchment areas. The hubs' centres were provided with adequate staff and services, including basic emergency obstetric and newborn care and 24-hour emergency services. All the hub centres were equipped with an ambulance. Staff were hired to fill the vacant sanctioned posts (funded from the district health budget), and additional staff were hired, paid from project funds.

In addition, from July 2008, Save the Children started a performance-based incentive (PBI) scheme, whereby all government-employed health facility workers were entitled to receive an additional 20-35% of their pay, according to performance criteria.

Staff hired directly by SC US were not entitled to incentives, but were paid a higher basic salary (43 staff were hired directly by SC US during the project lifetime--some 13% of the health workforce of the district).

The PBI component was designed around two measurement tools--one is a supervisory checklist, which was filled each month by an independent monitor (often from SC US), who checked on qualitative issues such as the hygiene of the facility, functionality of equipment, and maintenance of registers (see Table 1). The second was a set of targets set for preventive services, including coverage of antenatal care, deliveries by skilled birth attendants, post-natal care, newborn weighing, growth monitoring for under-threes, and three immunisation indicators (second maternal tetanus toxoid immunization (TT2) completed, infant immunisation started and immunisation completed). These were scored using information from the health management information system (HMIS). Table 2 illustrates how points were awarded in relation to these activities. Staff attendance records were also monitored.

**Table 1:** Supervision checklist and scorecard

S. No	Activity/Task	Observation	Total obtained
1	**Centre functional**	Open (5) Closed (0)	5

2	**Out-look** **of the Centre**	Poor (0) Satisfactory (1) Good (2) Excellent (3)	3

3	**Cleanliness** **of the centre**	I/C room (1) Pt. Waiting Area (1) LHV room(1) EPI room (1) Store (1)	5

4	**Staff uniform**	Yes (1) No (0)	1

5	**Necessary information display**	I/C (1) LHV (1) EPI (1)	3

6	**Attendance register maintained**	Yes (2) No (0)	2

7	**Staff leave record maintained**	Yes (1) No (0)	1

8	**Absent staff report submitted**	Yes No	If not, state reason

9	**Sufficient office furniture**	Available Not available	If not, state reason

10	**Diagnostic set**	Available Not available	If not, state reason

11	**Registers HMIS maintained**	OPD (1) EPI (1) Mother health (1) Child health (1) Birth register (1) Family planning (1) Stock register(1) Medicines register (1)	8

12	**OPD tickets** **(properly used)**	Yes (1) No (0)	1

13	**Last month HMIS report**	Complete (1) Incomplete (0)	1

14	**DEWS reports**	Submitted (1) Not submitted (0)	1

15	**Monthly staff meeting held**	Yes (1) No (0)	1

16	**Cold chain equipments**	Functional Non-functional	If not, state reason

17	**Vaccine availability**	Available Not available	If not, state reason

18	**Vaccine** **properly placed**	Yes (1) No (0)	1

19	**EPI Tech. following Monthly Tour Program**	Yes (1) No (0)	1

20	**EPI motor-cycle** **Log-book Maintained**	Yes (1) No (0)	1

21	**Delivery table** **available & clean**	Yes (1) No (0)	1

22	**Delivery kit** **available & clean**	Yes (1) No (0)	1

23	**Baby weighing machine available & functional**	Yes No	If not, state reason

24	**Physical Store verification**	Correct (1 No (0)	2

25	**Bin cards display**	Yes No	1

26	**X-ray**	Functional Non-functional	If not, state reason

27	**Laboratory**	Functional Non-functional	If not, state reason

	**Total**		40

**Table 2:** Performance assessment formula

PL registered for ANC			
Target	Achievement	%	Score

Expected pregnancies Catchment population/270	PL registered for ANC	Achievement/target x100	total = 10 IF > = 70,"10", IF > = 51,"8", IF > = 41,"6", IF > = 36,"4", IF > = 31,"3", IF > = 26,"2", IF > = 20,"1", IF < 20="0"

**PL completed TT2**			

Target	Achievement	%	score

Expected pregnancies catchment population/270	PL completed TT2	Achievement/target × 100	Total = 8 IF > = 60,"8", IF > = 51,"6", IF > = 41,"5", IF > = 36,"4", IF > = 31,"3", IF > = 26,"2", IF > = 20,"1", IF < 20="0"

**Deliveries by skilled birth attendants**			

**Target**	**Achievement**	**%**	**Score**

Expected deliveries catchment population/300	Deliveries by skilled birth attendants	Achievement/target × 100	Total = 10 IF > = 60,"10", IF > = 51,"8", IF > = 41,"6", IF > = 36,"4", IF > = 31,"3", IF > = 26,"2", IF > = 20,"1", IF < 20="0"

**Newborn weighed**			

**Target**	**Achievement**	**%**	**Score**

Total births catchment population/300	Newborn weighed	Achievement/target × 100	Total = 6 IF > = 60,"6", IF > = 55,"5", IF > = 46,"4", IF > = 38,"3", IF > = 30,"2", IF > = 20,1, IF < 20,"0"

**Post natal visits**			

**Target**	**Achievement**	**%**	**Score**

Deliveries in last month	Postnatal visits	Achievement/target × 100	Total = 6 IF > = 60,"6", IF > = 55,"5", IF > = 46,"4", IF > = 38,"3", IF > = 30,"2", IF > = 20,1, IF < 20,"0"

**Infants started immunization**			

**Target**	**Achievement**	**%**	**Score**

Infants in population 3.5/100 × patchment population	Infants started immunization	Achievement/target × 100	Total = 6 IF > = 81,"6", IF > = 65,"5", IF > = 50,"4", IF > = 35,"3", IF > = 20,2, IF < 20,"0"

**Infants completed immunization**			

**Target**	**Achievement**	**%**	**Score**

3.5/100 × Catchment population/12	Infants completed immunization	Achievement/Target × 100	Total = 8 IF > = 81,"8", IF > = 71,"7", IF > = 61,"6", IF > = 51,"5", IF > = 41,"4", IF > = 31,"3", IF > = 20,2, IF < 20,"0"

**Children < 3 weighed for growth monitoring**			

**Target**	**Achievement**	**%**	**Score**

11/100× Catchment Population/12	Children < 3 years weighed for GM	Achievement/Target × 100	Total = 6 IF > = 60,"6", IF > = 55,"5", IF > = 46,"4", IF > = 38,"3", IF > = 30,"2", IF > = 20,1, IF < 20,"0"

An overall weight of 40% was given to the 27 qualitative indicators and 60% to the 8 quantitative. According to the combined score reached, staff received a monthly supplement to basic pay of 20-35%, paid to all staff on the government payroll (which was managed in the district by SC US during the project duration). An average of 323 (between 320 and 415) health workers received performance based incentives over the project lifetime, paid direct into their bank accounts monthly.

As the project drew to a close in 2010, Save the Children US commissioned a review of the project, with particular emphasis on the PBI component.

## Methods

A mix of qualitative and quantitative research methods was used. Question guides were prepared for all of the qualitative research. For the quantitative, a framework of indicators guided the analysis.

The review was carried out in June 2010. Quantitative analysis was conducted of health management information system (HMIS) data, financial records, monthly progress reports, records of supervisory and performance scores of facilities, and project documents covering the period 2007 - mid-2010. In addition, eleven key informant interviews were carried out with stakeholders at SC US, the World Bank, provincial and district offices, and one local association.

The health facilities were chosen to represent the four hub areas, but also the stratification of performance: one was chosen from each of categories (very good, good, satisfactory and poor). At facility level, in-depth interviews were held with seven managers and other staff working at four facilities (three basic health units and one rural health centre). Eleven focus group discussions with staff (male and female) and community members (male and female) were also held. Data was collected by a team of three field researchers, together with the OPM consultant, while SC US provided one of their team members as a facilitator.

Analysis of quantitative data was undertaken using Excel. Qualitative reports were analysed thematically. The calculation of the performance indicators and of incentives changed after the first two months. Therefore the analysis omitted these two months so as not to bias trends, and covered July 2008-April 2010.

## Results

The findings are structured by a set of eight questions which should be asked of all pay-for-performance approaches. The first relates to design, and whether the targeted indicators were the right ones. Next we consider whether the system was well implemented. The third question is whether payments were in practice responsive to performance variation across the facilities. Fourth, did the payments motivate staff to change their behaviour, as was their primary goal? The fifth question is whether the approach was acceptable to the main local stakeholders. We then consider the core question of whether the PBI component improved overall performance of the health system. Evidence of possible perverse effects is also considered. Finally, we discuss the sustainability of the project.

### Did the PBI reward the right targets?

In terms of design, the use of two different scoring methods--one based broadly on 'process factors', which staff can directly influence (such as the cleanliness of the facility), and the other based on outputs, which are important but can only be partly influenced by supply-side actions--was seen by evaluators to represent a good balance. Average scores were higher for the supervision scores (73%) than the performance ones (46%), as performance indicators are 'stickier' and change more slowly (especially skilled deliveries, which are affected by important community beliefs, as well as cost and other access barriers). Differential thresholds for targets allowed for the fact that some indicators (e.g. ANC) started at much higher levels than others (e.g. facility deliveries).

**Figure 1:**
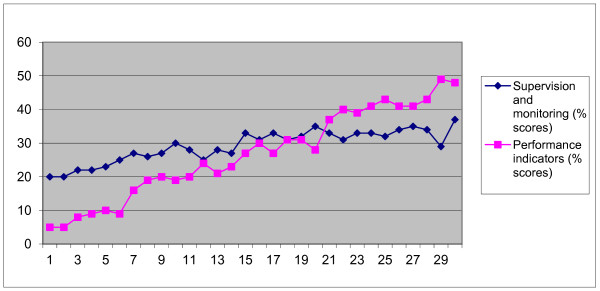
**Average performance and supervision scores, selected facilities, average for 2008-10**.

The two scores were correlated, as would be expected--generally, facilities with higher average supervision scores also had higher average performance scores, although the range was much greater for the latter (5%-48%), while supervision only spanned 20%-37% (see Figure 1).

### Was the PBI monitoring system well implemented?

The PBI component relied on monthly assessment by an independent monitor (often a SC US representative), based on observation and the facility registers. The process for measuring performance appears to have been reasonably regular for the Basic Health Units and Rural Health Centres, although there were months in which no assessment was made (and facilities received an automatic score, with staff receiving 20% incentives, which clearly undermines the approach). The average number of months for which supervisions were missed, per facility over the project lifetime, was 1.5, but for some facilities it was around one in three (10-12 months missed out of 30). The reasons given for missing supervision were either that the facility was under construction or that management attention was taken up for some major activity elsewhere. There were also some discrepancies between the overall score reached and the level of incentive paid, but these were limited.

The system worked less well for the civil dispensaries. All of the civil dispensaries scored less than 20 on the supervisory scores. The incentive paid to its staff never exceeded 20%. In addition, from the records it seems that the CDs were not visited regularly as part of the supervision and monitoring.

For the performance scores there was no independent verification of data taken from the facility registers.

### Were the PBI sufficiently responsive to changes in performance?

A successful PBI scheme (one which motivates individuals and teams) would be expected to produce positive trends in performance scores and positive trends in incentives. A change in ranking of individual facilities might also be expected over time, as facilities respond differentially to incentives. In Battagram, the supervision score component actually fell by 1 point (or -3%), reflecting its high starting point, while the performance score increased by 9 points (or 36%). However, the overall incentive score rose only by 2 points (7%) over the life of the project (comparing the first six months with the last six months), and payments to individual staff did not increase on average over time. This suggests that the overall project has been effective but that the link with the performance measurement system and incentives was weak. Some of the possible reasons for this are discussed in the section on motivation below.

**Figure 2:**
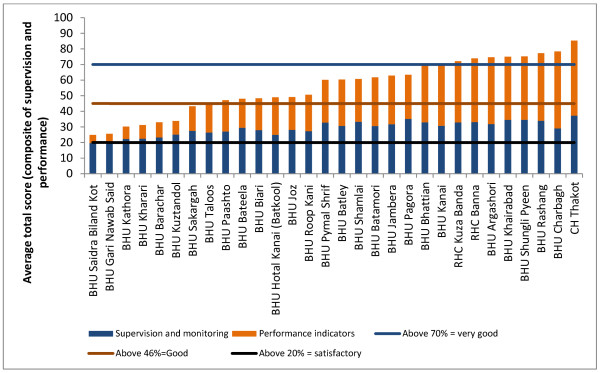
**Average total score for each basic health unit and rural health center (September 2008 - April 2010)**.

**Figure 3:**
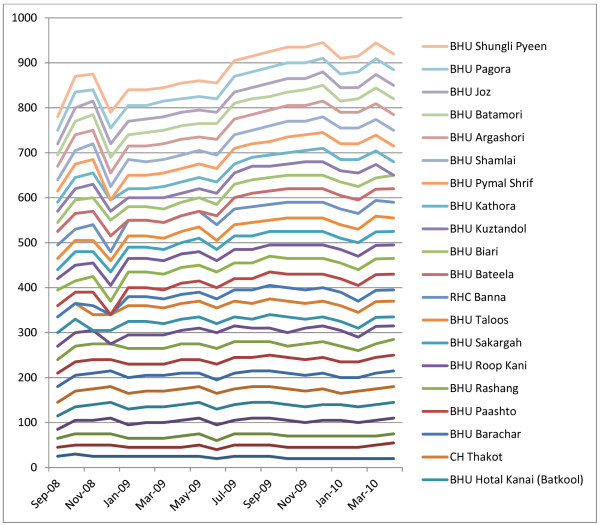
**Facility incentive scores, by month (September 2008 - March 2010)**.

On average, no facilities were graded as poor, and two-thirds fell within the incentive of 30%-35% band (see Figure 2), suggesting that the scale was not sufficiently sensitive (or that all facilities are really achieving on the same high level). Moreover facilities maintained more or less their position in relation to the starting point, and moved in synchronised patterns (see Figure 3). Those with higher performance at the start appear to have made more progress over time than those lower down. This indicates that prior features (either features relating to the services or to external factors such as the communities served) may have determined their performance.

### Did the PBI motivate health workers?

The structure of the incentives raises some questions in relation to their effectiveness in motivating higher performance. Under the current system, staff in a facility scoring a combined score of 0 would still receive an incentive of 20%. (Being absent without prior knowledge of the facility in charge was the only way to fail to achieve 20%.) In order to receive the additional 15%, their overall score would need to rise to 70% and above (see Table 3). Would that effort be justified? Interviews with staff suggested some scepticism, especially when the opportunity costs (no private practice) were considered. The government-hired senior staff lamented the fact that they were now not permitted to do private practice after work hours (which were 8 am to 2 pm). There was a general consensus amongst the facility staff that the incentives were not sufficient to cover the amount they had previously been making through private practice.

**Table 3:** Scoring for payment of incentives and the percentage incentive paid

Score	Ranking	Incentive
> 70%	Very good	35

46 to 70	Good	30

20 to 45	Satisfactory	25

< 20	Poor	20

Many staff were not aware of the detail of how the incentives were calculated. They were seen as a reflection of overall facility performance, rather than individual performance.

"I have no idea about any incentives. I only know that my salary has increased because I work hard." (Lady Health Visitor)

The average incentive paid was 29% of basic pay, and there was not much variation over time. In relation to gross pay, however, the proportion was lower--16% on average--and lower at basic health unit level (13%). This was commented on by staff, who requested a higher level of incentive (they suggested 50-100% of basic pay).

Some staff--those in district administration and in the TB centres--were paid incentives at a 'fixed rate' of 35%, while those hired by SC US direct were offered higher salaries and were not included in the PBI, although their performance was included in the overall rating of the facility. There was a general lack of understanding and transparency between these groups about each other's incentives and salary scales. The salary scale of the SC US staff was substantially higher than the government-hired staff--roughly equivalent to the government staff after the addition of 35% incentives, but both groups seemed unaware of this.

In absolute terms, PBI ranged from $15 per month for the lowest paid worker to $172 for the highest (the district director and deputy director of health). The average paid in monthly incentives was $48 per person.

### Were PBI acceptable to stakeholders?

Staff perception of PBI was positive--importantly, it was seen as being objective and as rewarding the performance of the whole facility. The fact that payments were made directly into staff bank accounts, and were proportionate to income, removed the element of individual discretion that can prove very corrosive in performance management schemes.

There were, however, some concerns in relation to equity--the main one related to the different treatment of staff hired by SC US, who were on a higher pay-scale and not included in the PBI scheme. The motivation behind this different treatment is not clear, but it does suggest that the PBI were being used primarily as a salary top-up for public servants.

Stakeholder feedback was positive about the project as a whole--communities particularly appreciated the low cost of services and the improvements to supply, including the availability of staff and medicines, and improvements in quality and appearance of the facilities. District and provincial managers were positive but were concerned about the longer term sustainability of the approach and how to eventually integrate it back into the system. Recommendations from the three main stakeholder groups included putting more emphasis on community-based activities, developing a closer relationship with the district and provincial authorities, particularly in relation to handing over the project, and providing more detailed feedback to staff on their performance, including discussion of how to improve it.

### Did the PBI improve performance?

**Figure 4:**
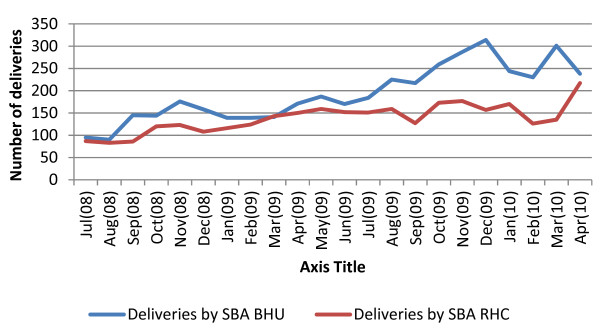
**Number of deliveries attended by skilled birth attendants, monthly, in basic health units and rural health centres from July 2008-April 2010, Battagram district**.

The review concluded that the project as a whole had contributed to an increase in the functionality of the health system and its outputs, as indicated by the interviews with staff and clients and also by the trends in specific services. Deliveries with skilled birth attendants, for example, increased by 150% between July 2008 and April 2010 (see Figure 4). Immunisation, while more variable month-by-month, still increased by 89% at basic health unit level, comparing the first six months of the project with the last six months. At rural health centres there was a reduction over the project lifetime--however, if this represents services shifting to the primary level, then that is an appropriate switch. Analysis of the tetanus typhoid uptake supports the view that users have been enabled to seek immunisation services at lower level facilities.

Comparison with district HMIS data from 2007 shows a substantial improvement in all indicators (see Table 4), with monthly outpatient visits, for example, increasing by more than 300% over the period.

**Table 4:** Trends in output indicators, 2007-10

	2007	2008	2009	2010	Percentage increase 2007-10
Average monthly outpatient visits	7029	20 568	33 550	28 274	302

Number registered for antenatal care	451	838	1223	1192	164

Number completed TT2 immunization	137	414	521	537	292

Deliveries assisted by skilled birth attendants	32	189	363	433	1252

Number of newborns weighed	1	124	301	414	41 250

Infants started immunization	1205	552	1014	1808	50

Children fully immunized	128	922	793	1692	1222

Family planning users	56	306	446	535	854

Robust attribution to the project requires longer term trend analysis, which was not undertaken as part of the review. However, comparing the multiple indicator cluster survey of 2001 with that of 2008, it can be seen that deliveries with skilled birth attendants had risen significantly at district and provincial level by the time of the introduction of the project, from 14% to 40.5% in Battagram and from 28% to 41% in the province as a whole. There are no comparable data for the other indicators.

Whether the increases can be attributed to the PBI component is in any case contentious. The PBIs represented 24% of the total project expenditure, and were accompanied by considerable additional investments in salaries, infrastructure, training, equipment and management support. The project as a whole ensured that there were adequate facility staff (including female medical officers), 24-hour emergency services, more equipment in the facilities (radiology and ultrasound), and a full range of immunisation, reproductive health and family planning services. Addressing the issue of costs to users, the ambulance service was provided free, as were medicines (which are now reliably stocked), delivery services (pre- and post-natal services), and the nutrition programme for under-fives and their mothers.

The case studies of individual facilities suggest that general investments in staffing and upgrading facilities were the main factors behind improved service delivery. Individual facilities show great fluctuations over time in performance scores, in particular, which are commonly linked with the availability (or absence) of key staff, such as doctors and nurses. The regular visits by the monitoring team could also have had a positive effect for some facility staff. The evaluations of the National Programme for Family Planning and Primary Health Care (2001 and 2008) found that regular visits by the supervisors where they carried out monitoring duties and the provision of supplies increased performance of lady health workers, as did continuing education.

### Did PBI cause any perverse effects?

A common concern with PBI-type approaches is that a focus on one set of indicators (in this case, preventive services) will squeeze out others. Analysis of total OPD visits over the project period reveal that utilisation rates rose from 0.42 per person per year (based on the first four months of the project) to 0.51 per person for the last four months. This is a rise of 22%, which is substantial, although still well below the WHO norm of 2 OPD visits per person per year. At the RHC level, the increase was from 1.13 to 1.85 per person per year - an increase of 63%. This suggests that in this respect at least, there were no perverse effects. There were however tensions created amongst staff in relation to the two different payment systems (one group receiving incentives, the other not), which reduced the motivation associated with the scheme.

### Sustainability of the approach

The project as a whole cost 184% of the district health expenditure, while the PBI element on its own was equivalent to 44% of the district health expenditure (see Table 5). Although the cost of the PBI element is low in USD per capita terms (USD 0.68 per person in the district per year), it is nevertheless high compared to the public spending of $1.65. The costs of the external monitoring which is required to support the PBI system have not been isolated but would also prove a barrier in scaling up or replicating this project. Stakeholders also expressed concerns about the sustainability of the project, given financial, managerial and organisational constraints in the public health sector.

**Table 5:** Total expenditure on project and on PBI (USD), Battagram district

	Total expenditure 2008-2010	Expenditure for one year	Per capita per annum spend	Ratio of project to government expenditure
Overall project	2 095 297	838 119	2.88	1.84

PBI component	497 103	198 841	0.68	0.44

Public expenditure on health in district	1 205 671	482 268	1.65	

Total	3 233 333	1 293 333	4.45	

## Discussion

The findings on this project raise issues which are specific to its design, implementation and context, but also broader reflections on some of the challenges of using pay-for-performance approaches.

It is generally accepted that professionals are motivated by the satisfaction of doing their jobs well (intrinsic motivation). Indeed, it is doubtful whether some valued-but-difficult- to-observe dimensions of quality (such as empathy or listening in the medical encounter) would be provided at all if physicians were solely interested in income. Thus, professionals have both non-monetary (that is, personal ethics, professional norms, regulatory control, clinical uncertainty) and monetary (from the payment system) incentives, all of which affect effort. It is possible that financial incentives may dilute professionals' intrinsic motivation. On the other hand, where health workers' pay is low in absolute terms, incentives may be an important channel to improve motivation through increasing their income levels. The effects of incentives on health worker motivation have been found to be very context-dependent in previous studies [5].

In the SC US project, the design does suggest that the PBI component was mainly functioning as a salary top-up, albeit with the need for staff to be physically present at facilities. In addition to basic salary came the basic incentives of 20%. The only margin for gain was the discretionary 15%, which was linked to general facility performance through a complex measurement system which most staff did not understand. The likelihood of individual motivation was therefore low, and most of the gains are likely to have come from general investments and the healthy balance of supply- and demand-side interventions which the project supported.

Paying for outputs (rather than for a composite index of quality measures and coverage targets) might have generated stronger incentives, though the risk of perverse effects might have been commensurately greater. These perverse effects might include neglecting unrewarded activities, distorting reporting systems to inflate coverage and staff moving to areas with higher performance or more favourable conditions for meeting targets.

One aim of paying for performance can be to encourage entrepreneurial behaviour amongst staff and managers. In this case, there was limited evidence of this, perhaps in part due to the low awareness by many staff members of exactly how the PBI scheme functioned. The existence of two tiers of staff--those hired directly by the NGO on higher salaries, and those on government staff with lower salaries but paid incentives--may also have weakened any motivational effects of the PBIs.

There is no consensus on how much PBI schemes should offer, in terms of additional resources, in order to motivate effectively. Clearly the level has to be set in context. However, in this case, the additional pay was below the opportunity costs in terms of private practice income foregone. In a tightly controlled project, it may be possible to ensure attendance and prevent staff from undertaking additional private practice, but in a less well managed environment, a low level of PBI might not fully achieve either goal. In other projects, where payment is made per output, the effectiveness of paying for performance has been linked to the payment per output, the effort required to deliver the output and the extent to which outputs are responsive to consumer versus provider decisions [6].

One challenge is the difficulty of designing a scheme which is complex enough to balance process and output measures, and to include a range of indicators to ensure that the system is not unduly focussed on a few interventions, and yet to be comprehensible to participants. The SC US project performed well in terms of design but less well in terms of simplicity. This will be a tension for all PBI processes. The weighting of the different indicators also involves a difficult judgement call, which in this case appeared to be made by the external agency alone, without much involvement of other stakeholders.

Another tension is that of rewarding team work versus the individual. In the case of this project, the measurement of performance focussed on team outputs, awarding the same incentives for all staff in a given facility, which was more acceptable, and yet pay went directly to individuals. This was appropriate for the setting and reduced tensions. The only individually assessed indicator was absenteeism--any member of staff absent without permission during the month was not eligible for incentives, which may have controlled the tendency to free-ride.

The review also supports wider evidence that there can be strongly demotivating effects where incentives are applied but not to all workers, so that there at least appear to be winners and losers. This reinforces the need for incentive strategies and combinations of incentives, rather than narrow incentives.

Another challenge is that individuals and facilities start at different levels of performance. This can be managed by setting individual targets, but these would have to be constantly adjusted in order to keep up with trends in performance, and ultimately high performers would be penalised for their more limited potential gains. In this case, targets were fixed for the group as a whole, which meant that certain facilities earned more from start to finish. Where this is linked to effort, this result would be seen as fair. However, it is more likely that the initial staffing position and other fixed factors determined facilities' performance.

It should also be noted that performance (in terms of coverage indicators) was assessed using facility data, which is amenable to manipulation, and was not independently verified or corroborated.

It is interesting that feedback from staff included the desire for more discussion of performance. A PBI approach might suggest that staff were already getting feedback in a very direct way, but in fact, the periodic checking of registers by an independent monitor, who then left without engaging with staff in discussion of how they had done, and why, and how it might be improved, was unsatisfactory from their point of view. This indicates the need to link 'objective' assessment systems with some more participatory forum, in which collective problem identification and solving can occur.

The whole nature of this particular PBI scheme was affected by the fact that it was implemented by an international non-governmental organisation with external funding, which was therefore able to provide independent and reliable third party systems for target-setting and assessment. The scale was also limited to one district, where the implementing organisation had strong on-the-ground presence. Where this function is internalised and scaled up, it will be much harder to maintain.

Another contextual issue is whether the post-disaster context of the area in Pakistan facilitated the acceptability of PBI. Some have argued that the evidence for effectiveness of PBI approaches is greater in post-conflict areas [7]] (which share features with post-disaster ones, in terms of a breakdown of infrastructure, at least, if not systems). In the case of Bhattagram, the main effect of emerging from disaster was that outputs were very low at the start of the project, so that the returns to general project investments could be commensurately large.

Clearly, it would have been desirable to quantify the cost-effectiveness of the PBI component in this project. However, that was not possible, for a number of reasons. First, the project outputs were many and varied--not easy to assimilate into one index. Secondly, any gains must be attributed jointly to government and project activities (and within the project, to PBI- and non-PBI elements). Thirdly, secular trends in growth (related to exogenous factors such as population and economic growth) must be allowed for in calculating gains. Finally, to judge the effectiveness of tying pay to performance requires that we distinguish between the motivational effects of higher pay per se, versus higher pay which is conditional on performance. With the data available, and in the absence of any control areas, these complex factors could not be adequately addressed.

## Conclusions

The review concluded that the SC US project in Battagram had contributed to rebuilding district health services. It did so at a cost of less than $4.5 per capita (combining project and district health expenditure) and achieved substantial growth in outputs. Staff, managers and clients were appreciative of the gains in availability and quality of services.

At the same time, the role that the PBI component played was less clear--PBI formed a relatively small component of pay, and did not increase in line with outputs. There was little evidence from interviews and data that the conditional element of the PBIs influenced behaviour. They were appreciated as a top-up to pay, but remained low in relative terms, and only slightly and indirectly related to individual performance. Moreover, they were implemented independently of the wider health system and presented a clear challenge for longer term integration and sustainability.

The PBI component nevertheless provided useful learning opportunities. It demonstrated that a transparent and objective process for measuring performance of a facility as a whole can be implemented in Pakistan without causing staff resentment. It demonstrated that a PBI approach focussed on preventive care can boost those services without reducing curative visits. It pioneered a 'scorecard' system which recognised the importance of process and output indicators. More generally, it has added to our understanding of how and in what circumstance PBI can contribute towards health sector goals.

## Competing interests

The authors declare that they have no competing interests.

## Authors' contributions

SW led on study design, analysis and drafting of the article; TZ managed local data collection and contributed to analysis and drafting; SJ led the qualitative research component and commented on drafts; AK and AB contributed to study design, data gathering and commented on drafts. All authors read and approved the final manuscript.
